# Comprehensive Assessment of Albumin and Uric Acid Levels in Oral Submucous Fibrosis: A Comparative Case-Control Study Involving Different Risk Groups

**DOI:** 10.7759/cureus.49811

**Published:** 2023-12-01

**Authors:** Amarshree A Shetty, Neha Gupta, Sonal Saigal, Ankur Bhargava, Debanti Giri, Animesh Mondal, Namrata Dagli, Dhaval Mehta

**Affiliations:** 1 Department of Paediatric and Preventive Dentistry, A.B. Shetty Memorial Institute of Dental Sciences, NITTE (Deemed to be University), Mangalore, IND; 2 Department of Oral Pathology, Microbiology, and Forensic Odontology, Dental College, Rajendra Institute of Medical Sciences (RIMS), Ranchi, IND; 3 Department of Oral Pathology and Microbiology, Hazaribag College of Dental Sciences and Hospital, Hazaribag, IND; 4 Department of Oral Medicine and Radiology, Dr. R. Ahmed Dental College and Hospital, Kolkata, IND; 5 Department of Oral and Maxillofacial Pathology, Dr. R. Ahmed Dental College and Hospital, Kolkata, IND; 6 Department of Dental Research, Karnavati Scientific Research Centre, Karnavati School of Dentistry, Karnavati University, Gandhinagar, IND; 7 Department of Oral Medicine and Radiology, Narsinhbhai Patel Dental College and Hospital, Sankalchand Patel University, Visnagar, IND

**Keywords:** areca nut, premalignant, tobacco, uric acid, serum albumin, oral submucous fibrosis

## Abstract

Background

Oral submucous fibrosis (OSMF) is a premalignant disorder that impacts the oral cavity and pharynx. Major risk factors for OSMF are attributed to the consumption of betel nuts or tobacco. These substances harbor various carcinogens that trigger the production of free radicals and reactive oxygen species. Antioxidants are pivotal in preserving cellular integrity and impeding the oncogenic transformation of body cells. In this context, albumin and uric acid, being primary antioxidants present in body fluids, bestow a defensive effect against this condition. Thus, the present study is designed to elucidate the differential concentration of albumin and uric acid between patient cases and healthy cohorts.

Methodology

This case-control study was conducted to evaluate the albumin and uric acid levels in individuals diagnosed with OSMF (cases) and compare them with healthy controls for a period of six months. A cohort of 100 individuals was partitioned into four groups, with each group comprising 25 individuals: Group I was made up of healthy individuals; Group II consisted of individuals who chew tobacco and areca nuts but are not affected by OSMF; Group III included individuals who only use tobacco without areca nuts and are afflicted with OSMF; and Group IV comprised individuals who consume a combination of areca nuts and tobacco and are diagnosed with OSMF. Biochemical evaluation was carried out using the BS-380 chemistry autoanalyzer (Mindray, Shenzhen, China), and the quantification of serum albumin and uric acid was performed by the uricase-peroxidase (POD) method with dihydroxybenzene sulfonic acid (DHBS).

Results

The study cohort of 100 individuals was made up of 70 males and 30 females, with an average age of 42.51 (11.62) years. The comparison of the mean concentration of serum albumin across all groups revealed that healthy controls exhibited the highest serum albumin concentration of 4.284 (0.618), with a statistically significant p-value (0.001) across all groups. A comparison of the mean value of serum uric acid among all groups showed that healthy controls had the highest value of serum uric acid (5.26±1.161), with a considerable p-value (0.001) between all groups.

Conclusion

The present study concluded that serum biomarkers assessed were high in healthy individuals and consumption of areca nuts, tobacco, and their products was significantly associated with low levels of albumin and uric acid. Therefore, both albumin and uric acid can be used as important biomarkers for uncovering oral premalignant lesions and conditions ahead of time and can also be used in mass screening.

## Introduction

Oral submucous fibrosis (OSMF) is a chronic premalignant condition afflicting the mouth and pharynx [1]. The characteristics of the disease include longstanding tenderness and undue collagen augmentation in deep connective tissues, which produces scars and fibrosis of tissues, mainly affecting the buccal mucosa [2,3]. It is the most common oral premalignant condition, with a malignant transformation rate of 1.9-9.13% [4,5]. Individuals in the second to fourth decades are most commonly affected, though it can occur at any age. The inception of this ailment is subtle and generally takes two to five years of time for the manifestation of symptoms. The Indian prevalence of this condition has been reported at 2.6% in males and 0.16% in females, and it mainly affects Southeast Asia and the Indian subcontinent [6]. The etiology of OSMF is multifactorial, in which autoimmunity, deficiencies of certain vitamins (B, C, and iron), areca nut/tobacco chewing, utilization of spicy foods, virus infection (human papillomavirus (HPV)), genetic mutations, and environmental, social, and behavioral factors are considered some of the prime etiological agents [6-8].

Betel quid is deemed a primary causative factor in the development and advancement of OSMF and oral cancer [9]. The International Agency for Research on Cancer posits that areca nut, a constituent of betel quid, functions as a Group I carcinogen in humans [10]. The areca nut possesses a plethora of alkaloids and polyphenol derivatives with carcinogenic capabilities; among them, arecoline is the most potent alkaloid present [11]. Extensive research has demonstrated that arecoline exhibits genotoxic and mutagenic properties that inhibit the proliferation of fibroblasts and keratinocytes in the oral and gingival mucosa. Carcinogens in areca nuts stimulate the generation of free radicals and reactive oxygen species [12].

Oxidative stress, a condition that arises when there is an upsurge of oxidants in the body, is described as an imbalance between peroxidants and antioxidants [9]. This stress promotes the production of reactive molecules like oxygen, hydrogen peroxide, and hydroxyl ions, precipitating DNA base modifications, strand fractures, and the damage of tumor suppressor genes. The human organism combats this detrimental effect through various protective measures, primarily through antioxidants present in body fluids like serum and saliva. Antioxidants are instrumental in neutralizing free radicals and preserving cellular integrity by preventing the malignant transformation of body cells. The provision of antioxidants can be controlled through both exogenous and endogenous mechanisms [13].

Reliable biomarkers are an urgent necessity as they can detect oxidative stress in its nascent stages [14,15]. Both uric acid and albumin are classified under the thiol group of antioxidants that neutralize free radicals [16]. Uric acid contributes to approximately 60% of the free radical scavenging capability [17,18]. The protein albumin, present in body fluids, plays a pivotal role in the etiopathogenesis of OSMF, and its deficiency is often observed in cases of oral malignancy [19-21]. The consumption of areca nuts and tobacco is a recognized risk factor that leads to a reduced blood supply and nutrient flow and is further linked with low levels of antioxidants. Both saliva and blood serve as mirrors of the physiological state of the human body, and albumin and uric acid act as vital investigative biomarkers for OSMF. Consequently, the current study was designed to measure albumin and uric acid in patients with OSMF and compare them with healthy individuals.

## Materials and methods

A case-control study was conducted to assess the albumin and uric acid levels among individuals with OSMF and controls over six months, from February to July 2023. Ethical approval was obtained from the AB Shetty Memorial Institute of Dental Sciences, Mangalore, India (approval number: ETHICS/ABSMIDS/395/2023). We got written permission from every study participant. Throughout the study, the anonymity and security of the data were upheld.

Eligibility criteria

Individuals with a history of chewing tobacco and/or areca nuts, patients diagnosed with OSMF who were habituated to areca nuts and tobacco, and healthy individuals were included. Exclusion criteria were set after taking a complete medical history: individuals having any systemic diseases (chronic liver disease, kidney diseases) that affect albumin levels in the body, chronic alcoholics (due to alternations in liver function and serum albumin and uric acid levels), individuals with weak immune systems, individuals undergoing radiotherapy and chemotherapy for cancer, and individuals who have been on certain medications like diuretics, steroids, and nonsteroidal anti-inflammatory drugs (NSAIDs) for the past few weeks.

Study population

The estimated effect size of 0.5, the significance threshold of 5%, and the 80% power to detect changes in serum albumin and the level of uric acid were all taken into consideration when determining the sample size based on statistical power analysis. This meant that there were 25 subjects in each group, for a total sample size of 100. A total of 100 individuals were recruited from the outpatient department (OPD) of the AB Shetty Memorial Institute of Dental Sciences. In total, there were four groups: Group I consisted of healthy cohorts; Group II consisted of individuals with a habit of chewing tobacco and areca nuts without OSMF; Group III comprised chewing tobacco users only without areca nuts affected by OSMF; and Group IV consisted of individuals chewing a combination of areca nuts and tobacco with OSMF.

Data collection

OSMF was evaluated as per the functional component [22]. To measure albumin and uric acid levels, all individuals were inculcated to starve one night before the data collection, and in the morning, 10 ml of blood was collected in a test tube by using disposable syringes. Ultracentrifugation was used to detach serum from other constituents of blood with care to prevent hemolysis, and the blood was stored at -200°C until analysis was performed. Biochemical analysis was performed using the BS-380 chemistry autoanalyzer (Mindray, Shenzhen, China), and assessment of serum albumin and uric acid was performed by the uricase-peroxidase (POD) method with dihydroxybenzene sulfonic acid (DHBS).

Statistical analysis

Values were entered into Excel sheets, and IBM SPSS Statistics for Windows, Version 26.0 (Released 2019; IBM Corp., Armonk, New York, United States) was utilized for the data study. A one-way analysis of variance (ANOVA) and Tukey's post-hoc test were applied to assess the mean value and mean difference of albumin and uric acid levels between groups.

## Results

The study sample had a mean age of 42.51±11.62 years with a 7:3 male-female ratio. The assessment of the mean concentration of serum albumin across all studied cohorts revealed that the highest concentration, 4.284±0.618, was observed in the healthy control group. Comparatively, the serum albumin concentrations within the cohorts of tobacco and areca nut consumers devoid of OSMF, areca nut consumers diagnosed with OSMF, and those consuming both areca nut and tobacco with OSMF were 3.644±0.396, 3.016±0.630, and 2.992±0.445, respectively, which was significant (Table 1).

**Table 1:** Comparison of serum albumin levels in all groups

Groups	Mean	SD	F value	p-value
Group I	4.284	0.618	33.080	0.001*
Group II	3.644	0.396		
Group III	3.016	0.630		
Group IV	2.992	0.445		

The analysis of the mean concentration of serum uric acid across all groups indicated that the control group again displayed the highest value, 5.26±1.161. The serum uric acid concentrations among tobacco and areca nut consumers without OSMF, areca nut consumers with OSMF, and those consuming both areca nut and tobacco with OSMF were 3.712±0.8590, 3.524±0.927, and 3.192±0.476, respectively (Table 2).

**Table 2:** Comparison of serum uric acid levels in all groups

Groups	Mean	SD	F value	p-value
Group I	5.26	1.161	26.505	0.001*
Group II	3.712	0.8590		
Group III	3.524	0.927		
Group IV	3.192	0.476		

The average distinction in serum albumin concentrations between Groups I and II, III, and IV was 0.640, 1.268, and 1.293, respectively, presenting a consequential p-value of <0.001. The average distinction in serum albumin levels between Groups II and I, III, and IV was -0.640, 0.628, and 0.652, respectively, with a p-value of <0.001. The average distinction in serum albumin between Groups III and I and II was -1.268 and -0.628, with statistically significant p-values, while no significant p-value was revealed in the mean difference between Groups III and IV. The mean difference in serum albumin levels between Groups IV and I and II was -1.292 and -0.652 with significant p-values, whereas the value between Groups IV and III was not significant (Table 3).

**Table 3:** Intergroup comparison of mean serum albumin levels NS: non-significant

Comparison of Group I with Groups II, II, and IV	Serum uric acid mean difference	Standard error	p-value	
Group II	0.640	0.150	0.001*	
Group III	1.268	0.150	0.001*	
Group IV	1.293	0.150	0.001*	
Comparison of Group II with Groups I, III, and IV				
Group I	-0.640	0.150		0.001*
Group III	0.628	0.150		0.001*
Group IV	0.652	0.150		0.001*
Comparison of Group III with Groups I, II, and IV				
Group I	-1.268	0.150		0.001*
Group II	-0.628	0.150		0.001*
Group IV	0.024	0.150		0.99 (NS)
Comparison of Group IV with Groups I, II, and III				
Group I	-1.292	0.150		0.001*
Group II	-0.652	0.150		0.001*
Group III	-0.024	0.150		0.99 (NS)

The mean variance in serum uric acid between Groups I and II, III, and IV was 1.548, 1.736, and 2.068, respectively, with a statistically significant p-value of <0.001. The mean variance in serum uric acid levels between Groups II and I was -1.548 with a highly significant p-value, whereas Groups II and III and IV had p-values of 0.188 and 0.520 with non-significant p-values. The mean variance in serum uric acid between Groups III and I was -1.736 with a statistically noteworthy p-value (<0.001), whereas the mean variance between Groups III and II and IV was -0.188 and 0.332, respectively, with no noteworthy p-values. The mean variance in serum uric acid between Groups IV and I was -2.068 with a statistically consequential p-value (<0.001), whereas the mean variance between Groups IV and II and III was -0.520 and -0.332 with non-significant p-values (Table 4).

**Table 4:** Intergroup comparison of mean serum uric acid levels NS: non-significant

Comparison of Group I with Groups II, II, and IV	Serum uric acid mean difference	Standard error	p-value
Group II	1.548	0.252	0.001*
Group III	1.736	0.252	0.001*
Group IV	2.068	0.252	0.001*
Comparison of Group II with Groups I, III, and IV			
Group I	-1.548	0.252	0.001*
Group III	0.188	0.252	0.878 (NS)
Group IV	0.520	0.252	0.173 (NS)
Comparison of Group III with Groups I, II, and IV			
Group I	-1.736	0.252	0.001*
Group II	-0.188	0.252	0.878 (NS)
Group IV	0.332	0.252	0.554 (NS)
Comparison of Group IV with Groups I, II, and III			
Group I	-2.068	0.252	0.001*
Group II	-0.520	0.252	0.173 (NS)
Group III	-0.332	0.252	0.554 (NS)

## Discussion

OSMF is a precancer state distressing the mouth and pharynx and has a link to an eminent risk of malignant transformation, but early diagnosis may give a better prognosis. Antioxidants, in essence, play a crucial role in scavenging free radicals and maintaining cell integrity by obstructing the malignant transformation of body cells [23]. With a wide range of antioxidants, the thiol group acts as the foremost scavenger of various free radicals. Among the numerous factors implicated in the development and progression of OSMF, the roles of albumin and uric acid levels in the blood have gained significant attention in recent years. Albumin, a key serum protein, plays a crucial role in maintaining oncotic pressure and is an indicator of nutritional status, while uric acid is a metabolic end product known to be influenced by various dietary and lifestyle factors. Betel nut/tobacco is considered a foremost threat factor for OSMF. Hence, in the present study, albumin and uric acid levels were measured and comparisons made between four groups.

The study population in the current research ranged from 21 to 61 years, which is in line with the study of Tiwari et al. [24]. Individuals in their middle ages (40-50 years) have likely been exposed to a myriad of risk factors over time. These risk factors can include lifestyle choices, environmental exposures, and genetic predispositions that cumulatively impact their oral health. Our study showed a greater percentage of males than females. This observation is in concordance with the studies of Reddy et al. [25] and Ali et al. [26].

The results indicate a clear association in serum albumin levels, with healthy controls having the highest values, followed by tobacco and areca nut users without OSMF, areca nut users with OSMF, and, finally, individuals using a combination of areca nut and tobacco with OSMF demonstrating the lowest serum albumin levels. This observation suggests a potential relationship between risk factors associated with OSMF and serum albumin levels, warranting further investigation into the etiology and pathophysiology of OSMF and its association with albumin metabolism. Iwao et al. [27] also found that albumin levels varied in different histological grades of oral cancer, with levels decreasing with higher grades. In 2010, Lawal et al. also reported that oral cancer had greater odds of occurring in subjects with low serum albumin levels [28]. Similarly, Metgud and Patel reported that oral leukoplakia and oral cancer patients are associated with low albumin levels in comparison with healthy subjects. According to various studies, serum albumin has a vital role in the early identification of oral premalignant lesions and conditions [29-31].

In the current investigation, the average concentration of serum uric acid demonstrated a superior level in the healthy control group relative to tobacco consumers and OSMF patients. Analogous observations were reported in the research conducted by Battino et al., documenting a decline in uric acid levels in patients suffering from a premalignant condition such as oral lichen planus (OLP) [32]. This finding aligns with the study by Dharmana et al., which illustrated a significant reduction in serum uric acid concentration in OLP patients compared to healthy subjects [33].

Shiva and Arab [34] and Sree and Kumar [35] have advanced the understanding that uric acid serves a crucial antioxidant role in human bodily fluids. They established that diminished serum uric acid concentrations directly correlate with the pathogenesis of OLP. Similarly, the studies conducted by Ara et al. [36] and Narang et al. [37] identified decreased concentrations of serum uric acid in instances of oral cancer. The initial trigger of OSMF is often attributed to the use of tobacco or areca nuts, which, in the long term, leads to impaired blood supply and nutrient inflow. This impairment is subsequently associated with reduced levels of antioxidants [38].

Differences in biomarkers could be attributed to various mechanisms. OSMF is known to involve chronic inflammation and oxidative stress. Inflammatory processes may lead to a decrease in albumin levels due to increased catabolism or impaired synthesis. Another mechanism is probably that nutritional status affects albumin levels. Reduced dietary intake or malabsorption due to oral mucosal lesions might lead to hypoalbuminemia. Additionally, uric acid levels are influenced by dietary factors, so nutritional status might impact both biomarkers.

TNF and IL-6, both known contributors to oral premalignant conditions and oral malignancies, have been observed to induce anorexia in affected individuals. In the context of the current investigation, the mean variance in serum albumin and uric acid concentrations across all four cohorts was examined. It was discerned that individuals who indulged in the simultaneous consumption of both areca nuts and tobacco exhibited lower levels in comparison to those who consumed either product in isolation. According to prior research, the occurrence rate of OSMF was notably higher among individuals who combined the consumption of areca nuts with one or more other products. The leachates from areca nuts are largely implicated in the progression of OSMF [39].

OSMF is a premalignant condition in which fibrosis starts due to limited blood and nutrient flow in associated areas, and over a long period, loss of muscle activity results due to complete fibrosis. This disease hampered the standard quality of life, and although a variety of measures exist for cure, the key step is to stop the habit [40,41]. Due to advancements in biomedical research, various new intervention strategies have been formulated for the prevention and management of these diseases. Antioxidants play a crucial role and are receiving more and more attention in the early recognition of oral premalignant conditions. As per various epidemiological studies, cell culture confirmed that the ingestion of antioxidants minimises the risk of cancer [29]. Therefore, this study was done to examine how serum albumin and uric acid were related to individuals who consumed areca or tobacco, a combination of both in OSMF, because early detection may help reduce the morbidity of oral cancers.

Limitations

Certain limitations in the present investigation warrant acknowledgment. There is potential for recall bias due to inaccuracies in participants' self-reported habits, which could compromise the integrity of the data. Conducted within a dental hospital environment, the findings of this study may not extend to populations with differing characteristics or those situated in diverse geographic locales. The inherent descriptive nature of the study precludes the establishment of cause-and-effect relationships.

## Conclusions

The case-control study concludes by underscoring that the consumption of tobacco and related products is associated with decreased levels of serum albumin and uric acid. These substances are deemed reliable biomarkers for the swift and early detection of oral premalignant conditions and lesions. Consequently, it is proposed that these biochemical examinations could be exceedingly beneficial for mass screening purposes. While this study was limited to a sample size of 100 subjects, it is recommended that future research involve a larger sample size to shed more light on the relationship between albumin and uric acid levels and OSMF.
